# Primary Results of the SALAMANDER Registry: A Multicenter Observational Cohort Study

**DOI:** 10.1161/JAHA.125.046625

**Published:** 2026-05-14

**Authors:** Wojciech Wańha, Łukasz Kuźma, Mariusz Kowalewski, Sylwia Iwańczyk, Joanna Grygier, Jan Jeske, Marco Frazzetto, Radosław Litwinowicz, Marian Burysz, Ewa Gaszewska‐Żurek, Claudio Sanfilippo, Tomasz Bochenek, Michał Lelek, Krystian Wita, Zenon Huczek, Agnieszka Kapłon‐Cieślicka, Tomasz Mazurek, Piotr Scisło, Jerzy Sacha, Krzysztof Struniawski, Brunon Tomasiewicz, Facehauwe Mezque, Wiktor Kuliczkowski, Krzysztof Bartuś, Sławomir Dobrzycki, Michał Święczkowski, Anna Kurasz, Paweł Kralisz, Dawid Miśkowiec, Jarosław D. Kasprzak, Marta Chamera, Maksymilian Mielczarek, Piotr Drewla, Rafał Gałąska, Fabrizio Ugo, Marco Franzino, Bernardo Cortese, Filippo L. Gurgoglione, Luigi Vignali, Giorgio Benatti, Federico Barocelli, Piotr Waciński, Piotr Niezgoda, Adam Sukiennik, Jacek Kubica, Mario Iannacone, Klaudia Kowalska, Łukasz Lewicki, Carmelo Grasso, Davide Capodanno, Fabrizio D'Ascenzo, Ovidio de Filippo, Francesco Bruno, Radosław Gocoł, Zbigniew Kalarus, Piotr Suwalski, Marek Grygier, Grzegorz Smolka, Gregory Y.H. Lip, Wojciech Wojakowski

**Affiliations:** ^1^ Department of Cardiology and Structural Heart Diseases Medical University of Silesia Katowice Poland; ^2^ Department of Cardiac Surgery and Transplantology National Medical Institute of the Ministry of the Interior and Administration Warsaw Poland; ^3^ DCB Academy Milan Italy; ^4^ Department of Invasive Cardiology Medical University of Bialystok Poland; ^5^ Thoracic Research Centre, Collegium Medicum, Nicolaus Copernicus University Innovative Medical Forum Bydgoszcz Poland; ^6^ Cardio‐Thoracic Surgery Department, Heart and Vascular Centre Maastricht University Medical Centre Maastricht The Netherlands; ^7^ First Department of Cardiology Poznan University of Medical Sciences Poznań Poland; ^8^ Harrington Heart and Vascular Institute University Hospitals Cleveland OH USA; ^9^ Department of Cardiovascular Surgery and Transplantology, Institute of Cardiology, Jagiellonian University Medical College St. John Paul II Hospital Krakow Poland; ^10^ Department of Cardiac Surgery Regional Specialist Hospital Grudziądz Poland; ^11^ Faculty of Medicine Bydgoszcz University of Science and Technology Bydgoszcz Poland; ^12^ Division of Cardiology Centro Cuore Morgagni Catania Italy; ^13^ First Department of Cardiology Medical University of Silesia Katowice Poland; ^14^ First Department of Cardiology Medical University of Warsaw Poland; ^15^ Department of Cardiology, University Hospital in Opole, Opole, Poland; Faculty of Physical Education and Physiotherapy Opole University of Technology Opole Poland; ^16^ Department of Heart Diseases Wroclaw Medical University Wroclaw Poland; ^17^ Department of Cardiology Medical University of Lodz Poland; ^18^ First Department of Cardiology Medical University of Gdansk Poland; ^19^ Division of Interventional Cardiology Sant'Andrea Hospital Vercelli Italy; ^20^ Division of Cardiology Parma University Hospital Parma Italy; ^21^ Clinical Department of Interventional Cardiology Medical University of Lublin Poland; ^22^ Department of Cardiology and Internal Medicine Collegium Medicum, Nicolaus Copernicus University Bydgoszcz Poland; ^23^ U.O. Cardiologia Interventistica, Ospedale San Giovanni Bosco, ASL Città di Torino Torino Italy; ^24^ Kashubian Center for Heart and Vascular Diseases Wejherowo Poland; ^25^ Division of Cardiology, Azienda Ospedaliero‐Universitaria Policlinico “G. Rodolico ‐ San Marco” University of Catania Italy; ^26^ Division of Cardiology, Cardiovascular and Thoracic Department A.O.U. Città della Salute e della Scienza Turin Italy; ^27^ Department of Medical Sciences University of Turin Italy; ^28^ Department of Cardiac Surgery, Upper‐Silesian Medical Centre Medical University of Silesia Katowice Poland; ^29^ Department of Cardiology, DMS in Zabrze Medical University of Silesia Katowice Poland; ^30^ Department of Cardiology, School of Health Sciences (SHS) Medical University of Silesia Katowice Poland; ^31^ Liverpool Centre for Cardiovascular Science, University of Liverpool Liverpool John Moores University and Liverpool Heart and Chest Hospital Liverpool UK; ^32^ Danish Center for Health Services Research, Department of Clinical Medicine Aalborg University Aalborg Denmark; ^33^ Department of Cardiology, Lipidology and Internal Medicine with Intensive Coronary Care Unit Medical University of Bialystok Poland

**Keywords:** antithrombotic regimen, left atrial appendage occlusion, procedural success, technical success, Atrial Fibrillation, Anticoagulants

## Abstract

**Background:**

The balance between thromboembolic complications and bleeding risk in anticoagulated patients with atrial fibrillation remains challenging, with left atrial appendage occlusion (LAAO) representing a potential alternative. Our prospective multicenter study aimed to evaluate the feasibility, safety, technical, and procedural outcomes of the contemporary practice of stand‐alone LAAO.

**Methods:**

The SALAMANDER (Stand‐Alone Left Atrial Appendage Occlusion for Thromboembolism Prevention in Nonvalvular Atrial Fibrillation Disease) registry is a real‐world, multicenter, observational cohort study conducted at 16 cardiac centers in Europe between 2010 and 2024, evaluating the safety and efficacy of LAAO using contemporary devices.

**Results:**

A total of 1660 patients were enrolled, with a median age of 76 (interquartile range, 70–81) years and 38% being women. The median CHA_2_DS_2_‐VASc score was 4 (interquartile range, 3–5). The most common indication for LAAO was significant bleeding (83.7% of patients), most frequently during treatment with direct oral anticoagulants (68.8%) more than vitamin K antagonists (24.9%). The predominant bleeding sites were the lower (27.9%) and upper (21.7%) gastrointestinal tracts, with 17.4% having a history of hemorrhagic stroke. The most common antithrombotic regimen before the procedure was direct oral anticoagulants (48.9%), while postprocedural therapy most often included dual antiplatelet therapy (50%). The technical success rate was 95.5%, with residual leak (2.2%) and tamponade (1.4%) as the main causes of failure. Procedural success was 90.5%, most often limited by vascular complications (3.7%), periprocedural death (1.1%), and major bleeding (0.7%). Technical and procedural success rates did not differ significantly between the devices used.

**Conclusions:**

In this large prospective cohort, technical and procedural success rates were similar across all LAAO devices, suggesting comparable safety and efficacy. Postprocedural therapy typically involves dual antiplatelet therapy, with all patients requiring some pharmacological treatment.

**Registration:**

URL: https://www.clinicaltrials.gov; Unique identifier: NCT05144958.

## Graphical abstract

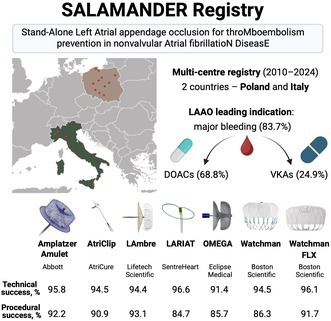

**Nonstandard Abbreviations and Acronyms**
ADALA: Low‐Dose Direct Oral Anticoagulation Versus Dual Antiplatelet Therapy After Left Atrial Appendage Occlusion
DAPT: dual antiplatelet therapy
EWOLUTION: Registry on WATCHMAN Outcomes in Real‐Life Utilization
LAA: left atrial appendage
LAAO: left atrial appendage occlusion
LOGIC: Left Atrial Appendage Occlusion in Patients With Gastrointestinal or Intracranial Bleeding
OAC: oral anticoagulation
PRAGUE‐17: Left Atrial Appendage Closure Versus Novel Anticoagulation Agents in Atrial Fibrillation
PREVAIL: Obicetrapib and Cardiovascular Outcomes: A Placebo‐Controlled, Double‐Blind, Randomized Phase 3 Study to Evaluate the Effect of 10 mg Obicetrapib in Participants With Atherosclerotic Cardiovascular Disease Who Are Not Adequately Controlled Despite Maximally Tolerated Lipid‐Modifying Therapies
PROTECT AF: WATCHMAN Left Atrial Appendage System for Embolic Protection in Patients With Atrial Fibrillation
SALAMANDER: Stand‐Alone Left Atrial Appendage Occlusion for Thromboembolism Prevention in Nonvalvular Atrial Fibrillation Disease
SAPT: single antiplatelet therapy
VKA: vitamin K antagonists

**Research Perspective**

**What Is New?**

- In the large multicenter SALAMANDER (Stand‐Alone Left Atrial Appendage Occlusion for Thromboembolism Prevention in Nonvalvular Atrial Fibrillation Disease) registry, stand‐alone left atrial appendage occlusion using 7 contemporary devices was associated with high technical (95.5%) and procedural (90.5%) success rates, with no meaningful differences between devices.
- Postprocedural antithrombotic therapy in SALAMANDER was heterogeneous, with antiplatelet‐based regimens most commonly used at discharge.

**What Question Should Be Addressed Next?**

- Prospective studies are needed to define optimal post‐left atrial appendage occlusion antithrombotic strategies that minimize bleeding while maintaining protection from device‐related thrombosis and thromboembolic events.

Atrial fibrillation (AF) remains the most common sustained cardiac arrhythmia, affecting an estimated 52.55 million individuals globally, with its prevalence expected to rise further due to population aging and increasing burden of comorbidities.^1^ The left atrial appendage (LAA) is the most frequent source of thrombotic material in patients with nonvalvular AF, leading to cerebrovascular events.^2^ Thus, current guidelines recommend oral anticoagulation (OAC), including both vitamin K antagonists (VKAs) and direct oral anticoagulants (DOACs), as the cornerstone of thromboembolic prophylaxis in patients with AF with elevated stroke risk, as assessed by the CHA_2_DS_2_‐VASc/CHA_2_DS_2_‐VA scores.^3^, ^4^ While OAC therapy has proven efficacy in reducing stroke risk, its use is often limited by bleeding complications, drug–drug interactions, renal impairment, frailty, or reduced compliance.^5^ Therefore, a significant proportion of patients with AF either do not receive OAC or discontinue therapy prematurely, leaving them exposed to substantial thromboembolic risk.^6^, ^7^

In the past decade, left atrial appendage occlusion (LAAO) has been established as a valid and safe alternative for this group of patients.^8^, ^9^ Based on the 2024 European Society of Cardiology guidelines on AF,^3^ percutaneous LAAO may be considered in patients with AF and contraindications for long‐term anticoagulant treatment to prevent ischemic stroke and thromboembolism. In the PROTECT AF (WATCHMAN Left Atrial Appendage System for Embolic Protection in Patients With Atrial Fibrillation) and PREVAIL (Obicetrapib and Cardiovascular Outcomes: A Placebo‐Controlled, Double‐Blind, Randomized Phase 3 Study to Evaluate the Effect of 10 mg Obicetrapib in Participants With Atherosclerotic Cardiovascular Disease Who Are Not Adequately Controlled Despite Maximally Tolerated Lipid‐Modifying Therapies) randomized clinical trials, LAAO using the Watchman device (Boston Scientific, Natick, MA) was noninferior to warfarin in preventing stroke, systemic embolism, and cardiovascular death, with a favorable long‐term safety profile.^10^, ^11^, ^12^ Furthermore, in the PRAGUE‐17 (Left Atrial Appendage Closure Versus Novel Anticoagulation Agents in Atrial Fibrillation) trial, LAAO was noninferior to DOACs in preventing major AF‐related cardiovascular, neurological, and bleeding events.^12^ Importantly, LAAO technology has made substantial progress with less traumatic and more patient‐specific adaptive devices, which have improved the safety and efficacy of LAAO.

Postprocedural antithrombotic management following LAAO remains an unresolved challenge. Even patients undergoing the procedure due to contraindications to OAC often require temporary postimplantation therapy that carries similar bleeding risks. While guidelines based on randomized clinical trial protocols mention 45 days of a VKA plus aspirin, followed by dual antiplatelet therapy (DAPT) and then aspirin monotherapy (if there are no major peridevice leaks), real‐world strategies vary widely.^13^ Epicardial LAAO may offer an additional advantage in truly OAC‐ineligible patients, as it typically avoids the need for any postprocedural anticoagulation altogether.

This prospective, multicentre registry study aimed to assess the feasibility, safety, technical, and procedural outcomes of stand‐alone LAAO (via fully thoracoscopic‐epicardial, percutaneous‐endocardial, or hybrid endo‐epicardial access) for thromboembolism prevention in patients with nonvalvular AF.

## Methods

The data that support the findings of this study are available from the corresponding author upon reasonable request.

### Study Design

The SALAMANDER registry is a real‐life, multicenter, observational study conducted across 16 high‐volume cardiac centers in Europe, with the primary objective of evaluating the safety and efficacy of LAAO using a variety of currently available technologies.^14^ Overall, 7 distinct devices have been assessed within the registry framework, representing the technological diversity of current clinical practice. These include (1) AtriClip (AtriCure, Mason, OH), a fully thoracoscopic, epicardial clip system applied externally to occlude the LAA via minimally invasive surgical access; (2) Watchman (Boston Scientific), a percutaneous, endocardial occluder delivered transseptally to seal the LAA from within the atrium; (3) Watchman FLX (Boston Scientific), the next‐generation version of the Watchman device, with enhanced conformability and anchoring features; (4) Amulet (Abbott, Plymouth, MN), a dual‐seal endocardial device comprising a lobe and a disc, suitable for complex LAA morphologies; (5) LARIAT (SentreHeart, Redwood City, CA), a hybrid endo‐epicardial system involving transseptal endocardial guidance and epicardial suture‐based ligation; (6) Lambre (Lifetech Scientific, Shenzhen, China), a self‐expanding, endocardial occluder with a distinctive anchoring mechanism and adaptability to varying appendage anatomies; and (7) Omega (Shape Memory Medical Inc., Santa Clara, CA), an endocardial device using shape memory.

The patients' data were collected using an electronic case report form developed by the Polish Cardiac Society and were protected in accordance with the applicable Polish regulations, the General Data Protection Regulation, and institutional standard operating procedures (Data S1). The study was conducted in line with the principles of the Declaration of Helsinki and was registered at www.ClinicalTrials.gov (NCT05144958). The study adhered to Strengthening the Reporting of Observational Studies in Epidemiology guidelines for observational studies (Table S1). Ethical approval was obtained from the local Bioethics Committee of the Upper‐Silesian Medical Centre of the Silesian Medical University in Katowice, Poland (PCN/CBN/0052/52/139/I/22). Due to the noninterventional, observational nature of this prospective registry, the requirement for informed consent was waived. To minimize potential bias, all participating centers followed standardized data collection procedures with predefined event definitions and protocol‐based measures to ensure cross‐center consistency in reporting. In addition, regular data quality checks, source data verification, and central data validation were performed to ensure the completeness, accuracy, and uniform ascertainment of outcomes across sites.

### Study Population

**Figure 1:**
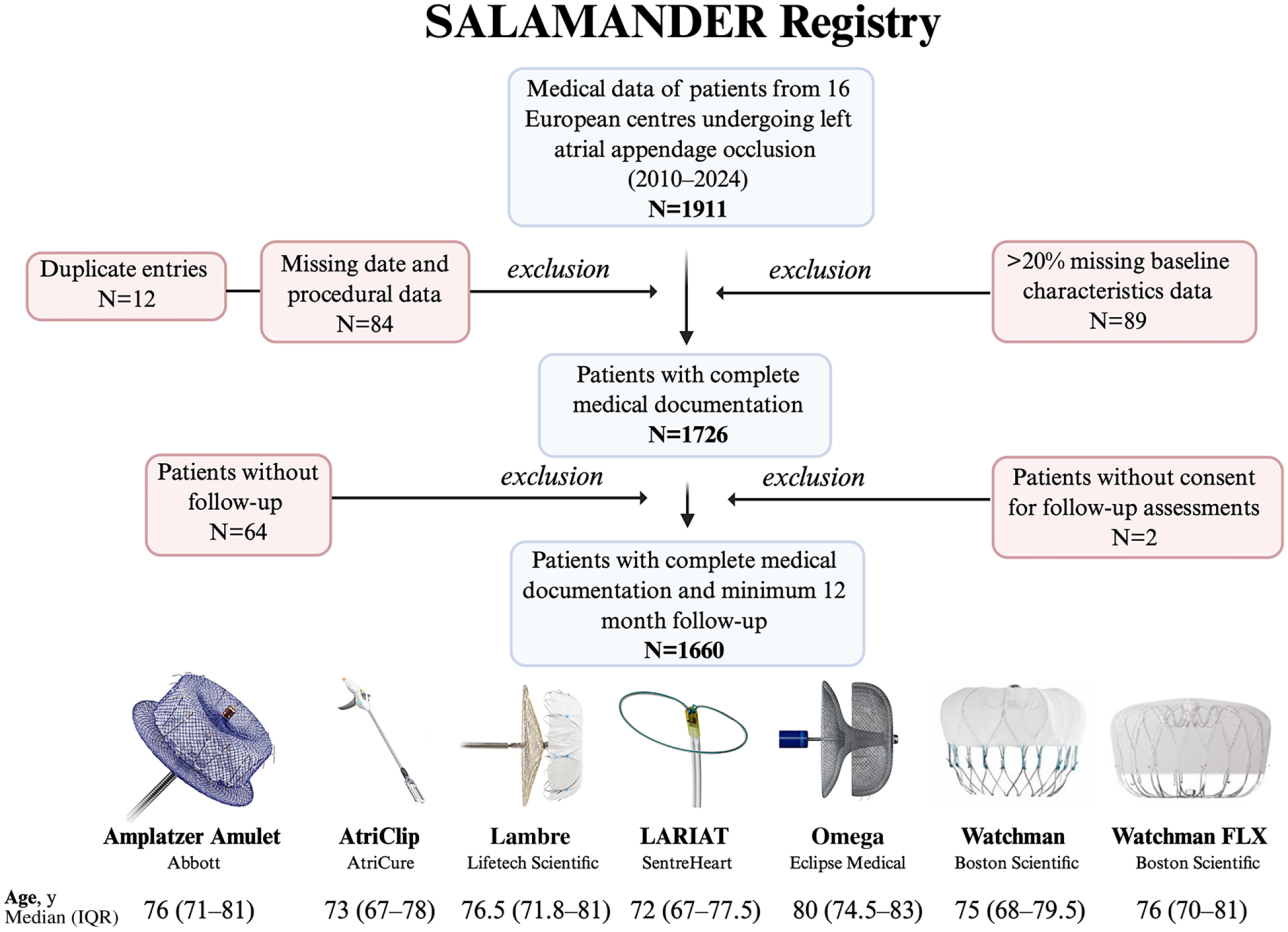
Flowchart of the study design. IQR indicates interquartile range.

Patients in the study have been continuously recruited at each participating center to ensure unbiased inclusion of the eligible population. The enrollment period for this analysis spanned from October 29, 2010, to December 19, 2024. Inclusion criteria for the SALAMANDER registry comprise the following: (1) age ≥18 years; (2) diagnosis of paroxysmal, persistent, or permanent nonvalvular AF; (3) increased thromboembolic risk, defined as a CHA_2_DS_2_‐VASc score ≥2 in men or ≥3 in women; (4) elevated bleeding risk, particularly in patients with a HAS‐BLED score ≥3; (5) history of significant bleeding events; (6) documented bleeding complications while on OAC, including VKAs; (7) presence of comorbid conditions predisposing to bleeding, such as coagulopathies, thrombocytopenia, or end‐stage renal disease requiring dialysis; and (8) failure of oral anticoagulation due to stroke recurrence or associated cerebrovascular abnormalities such as cerebral aneurysm. Participants in this registry met all the inclusion criteria and did not meet any exclusion criteria. Study sites are listed in Table S2. The flowchart of the study design is presented in Figure 1.

### End Point Definitions

Clinical and procedural data are collected in accordance with the study protocol and predefined criteria for success and complications, thereby enabling a robust assessment of the safety and efficacy of LAA closure in real‐world clinical practice. The primary outcomes analysis focused exclusively on clinical events occurring during the index hospitalization (from the LAAO procedure until hospital discharge). Imaging outcomes were additionally assessed at a 1‐time control visit within 3 months after the procedure. This study uses structured, prespecified end points based on the definitions outlined in the Munich Consensus.^15^ As the SALAMANDER registry is ongoing, postdischarge clinical outcomes are not analyzed in this article, which focuses on baseline characteristics, procedural data, and in‐hospital outcomes. Longitudinal follow‐up outcomes (24 months) will be reported separately once the full data set becomes available to avoid bias related to unequal follow‐up duration and potential temporal changes in device selection and procedural strategies.

The primary end point was technical success, defined as complete LAA exclusion without device‐related complications and with no residual peridevice leak >5 mm. Procedural success was defined as achievement of technical success without major periprocedural complications, including vascular injury, thromboembolic events (eg, stroke or air embolism), periprocedural death, or major bleeding. Major bleeding was defined as type 3 or higher according to the Bleeding Academic Research Consortium criteria. Ischemic stroke was defined as an acute focal neurological deficit presumed to be due to ischemia, persisting ≥24 hours, or <24 hours with imaging confirmation of infarction. Transient ischemic attack was defined as a rapid‐onset focal neurologic deficit due to cerebral ischemia, resolving within 24 hours. Systemic embolism was defined as an occlusive thrombotic event in a systemic artery with clinical manifestations. Effective device occlusion is defined as a residual jet ≤5 mm, evaluated intraoperatively or during control visit (transoesophageal echocardiography or computed tomography), depending on the specific device used. Given the heterogeneous nature of available LAAO technologies, including 7 distinct occluder systems—Watchman, Watchman FLX, Amulet, LARIAT, AtriClip, Lambre, and Omega—the study adopted uniform, device‐agnostic definitions of success. Despite differences in design, deployment strategy (endocardial, epicardial, or hybrid), and imaging control protocols, the endpoint definitions were standardized across all devices to enable valid interdevice comparisons. Procedural metrics, including implantation depth, compression ratio, residual leak, need for partial or full recapture, and imaging features (eg, spontaneous echo contrast, coverage of trabeculations), were systematically collected. Device‐specific parameters were reported using a harmonized data structure, and all echocardiographic data were evaluated by a central core laboratory to ensure consistency across the diverse technologies.

### Statistical Approach

Data were summarized using mean±SD and median (interquartile range) for continuous data, and count (percentage) for categorical data. Continuous variables were tested for distribution by the Kolmogorov–Smirnov test. For comparisons between 2 groups, the unpaired Student's *t* test or Mann–Whitney *U* test was applied, depending on data distribution. For comparisons across >2 groups, ANOVA or the Kruskal–Wallis test was used as appropriate. Categorical variables were compared using the χ^2^ test or Fisher's exact test. To identify independent predictors of procedural success and clinical outcomes, multivariable logistic regression analysis was performed. Variables with a *P* value <0.05 in univariable analysis were included in the multivariable model. Results are expressed as odds ratios with 95% CIs. Figures were created in BioRender (https://biorender.com/). Given the minimal extent of missingness and the fact that these variables were not used for building adjusted multivariable models at this stage of the analysis, no imputation procedures were performed.

## Results

### Characteristics of Participants

The study enrolled 1660 patients (median age, 76 [interquartile range, 70–81] years; 38% women). A total of 98.7% of patients had AF, and the remaining 1.3% were diagnosed with atrial flutter. The median LVEF was 55%. The most common comorbidities were hypertension (90%), chronic kidney disease (49.9%), and coronary artery disease (37.7%). A prior history of ischemic stroke was reported in 26.3% of patients and systemic embolic events in 12.1%. The median CHA_2_DS_2_‐VASc was 4 (interquartile range, 3–5). The median HAS‐BLED score was 4 (interquartile range, 3–4). Detailed characteristics of enrolled participants are presented in Table 1.

**Table 1:** Study Group Characteristics

Variables	Observations	Value
Baseline characteristics		
Age, y, median (IQR)	1660	76 (70–81)
Female sex, N (%)	1660	630 (38)
Length of hospital stay, median (IQR)	1660	4 (3–5)
Body mass index, median (IQR)	1322	27.1 (24.2–30.1)
AF, N (%)	1660	1638 (98.7)
Atrial flutter, N (%)	1660	22 (1.3)
Left ventricular ejection fraction, %, median (IQR)	1660	55 (46–60)
European Heart Rhythm Association class ≥2a, N (%)	1616	393 (24.3)
New York Heart Association class ≥2, N (%)	771	577 (74.8)
Hypertension, N (%)	1660	1494 (90)
Diabetes, N (%)	1660	562 (33.9)
Lung diseases, N (%)	1660	104 (6.3)
Mitral stenosis (moderate or greater), N (%)	1660	9 (0.5)
Mitral regurgitation (moderate or greater), N (%)	1660	345 (20.8)
Aortic stenosis (moderate or greater), N (%)	1660	100 (6)
Aortic regurgitation (moderate or greater), N (%)	1660	60 (3.6)
Chronic kidney disease, N (%)	1660	689 (49.9)
Prior ischemic stroke, N (%)	1660	437 (26.3)
Prior systemic embolism, N (%)	1660	201 (12.1)
Coronary artery disease, N (%)	1660	626 (37.7)
Prior myocardial infarction, % (N)	1660	339 (20.4)
Peripheral artery disease, N (%)	1660	331 (18.7)
Carotid artery disease, N (%)	1660	180 (10.8)
CHA_2_DS_2_VASc score, median (IQR)	1660	4 (3–6)
CHA_2_DS_2_VA score, median (IQR)	1660	4 (3–5)
HAS‐BLED score, median (IQR)	1660	4 (3–4)
Baseline hemoglobin, g/dL, median (IQR)	1287	12.5 (11–14)
Baseline glomerular filtration rate, mL/min per 1.73 m^2^, median (IQR)	1550	61 (43–77.8)
Baseline platelet count, median (IQR)	1281	201 (161–253)
Indications		
Clinically significant prior bleeding	1660	1389 (83.7)
Anticoagulation type		
Bleeding on VKA	1389	346 (24.9)
Bleeding on DOAC	1389	955 (68.8)
Bleeding on low‐molecular‐weight heparin	1389	28 (2)
Bleeding on antiplatelet therapy	1389	19 (1.4)
Bleeding without therapy	1389	42 (3)
Bleeding site		
Unidentified bleeding site	1389	166 (12)
Respiratory system	1389	26 (1.9)
Urinary system	1389	77 (5.5)
Ocular	1389	35 (2.5)
Lower gastrointestinal tract	1389	387 (27.9)
Intracranial hemorrhage	1389	242 (17.4)
Mucocutaneous bleeding	1389	170 (12.2)
Upper gastrointestinal tract	1389	301 (21.7)
Other	1389	168 (12.1)
Prior blood transfusion	1389	524 (37.7)
Coagulopathies	1660	35 (2.1)
Failure of OAC	1660	93 (5.6)
Amyloid angiopathy	1660	16 (1)
Liver cirrhosis	1660	6 (0.4)
Allergic reactions	1660	16 (1)
Cerebral aneurysm	1660	25 (1.5)
Thrombocytopenia	1660	25 (1.5)
Dialysis	1660	68 (4.1)
Other	1660	140 (8.4)
Procedural characteristic		
Watchman FLX, N (%)	1660	648 (39%)
Watchman, N (%)	1660	291 (17.5%)
Amplatzer Amulet, N (%)	1660	500 (30.1%)
Lambre, N (%)	1660	72 (4.3%)
LARIAT, N (%)	1660	59 (3.6%)
Omega, N (%)	1660	35 (2.1%)
AtriClip, N (%)	1660	55 (3.3%)
Intraoperative parameters		
General anesthesia, N (%)	1660	928 (55.9%)
Anesthesia with intubation, N (%)	1660	541 (32.6%)
Procedure duration, min, mean±SD	1277	66.2 (28.6)

AF indicates atrial fibrillation; DOAC, direct oral anticoagulant; IQR, interquartile range; OAC, oral anticoagulation; and VKA, vitamin K antagonist.

Patient characteristics according to the implanted device are presented in Table S3. The most common types of LAA shapes were chicken wing (63.5%), windsock (13.5%), and cauliflower (12.5%). Additional data on the characteristics of LAA are presented in Table S4.

### Indications for LAAO

The most common indication for LAAO was significant bleeding, in 83.7% of enrolled patients. Among these bleeding events, 68.8% occurred during treatment with DOACs and 24.9% during therapy with VKAs. The most frequent bleeding sites were the lower (27.9%) and upper (21.7%) gastrointestinal tracts, and 17.4% had a history of hemorrhagic stroke. The second‐ and third‐most‐common indications were other reasons (8.4%) and failure of OAC (5.6%), respectively. A detailed description of the indications for LAAO is presented in Table 1.

### Procedural Characteristic

The most frequently used devices were Watchman FLX (39%), Amplatzer Amulet (30.1%), and Watchman (17.5%). General anesthesia was administered to 55.9% of enrolled participants, of which 32.6% received intubated anesthesia. The remaining procedural characteristics are presented in Table 1.

### Antithrombotic Regimen

Before the procedure, 91.7% of patients received OAC in the form of DOACs (48.9%), VKAs (17.1%), DOACs with single antiplatelet therapy (SAPT; 12%), triple antithrombotic therapy (5.7%) or low‐molecular‐weight heparin (4.1%).

**Figure 2:**
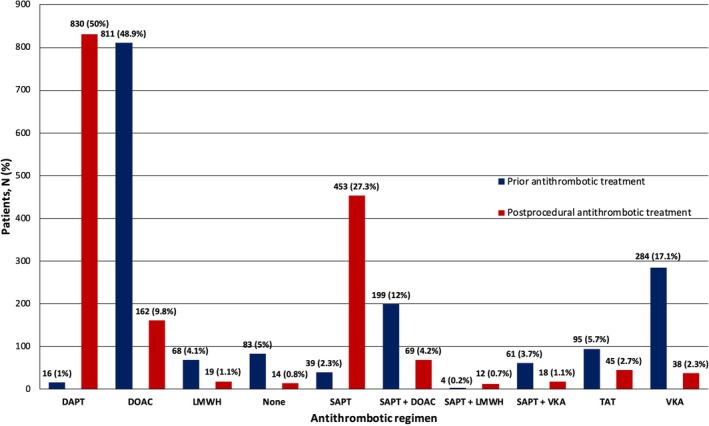
Antithrombotic regimens before and after the procedure. DAPT indicates dual antiplatelet therapy; DOAC, direct oral anticoagulant; LMWH, low‐molecular‐weight heparin; SAPT, single antiplatelet therapy; TAT, triple antithrombotic therapy; and VKA, vitamin K antagonist.

The most common antithrombotic regimens after the procedure were DAPT (50%), SAPT (27.3%), and DOACs (9.8%). The percentage distribution of various antithrombotic regimens is presented in Figure 2.

The most common antithrombotic regimen before the procedure, DOACs (48.9%), was most often switched to DAPT (45.4% of all DOAC‐treated patients) or SAPT (24.4% of all DOAC‐treated patients) postprocedurally, while in 18.8% of cases it was continued. Among patients receiving VKAs (17.1% of all patients before the procedure), 58.8% were switched to DAPT, 22.2% to SAPT, and 12% continued their previous regimen. The majority of patients receiving low‐molecular‐weight heparin before the procedure (4.1% of all patients) were switched to DAPT after the LAAO (70.6% of all low‐molecular‐weight heparin–treated participants). Among patients receiving triple antithrombotic therapy before the procedure, 65.3% were treated with DAPT and 31.6% with SAPT following LAAO.

**Figure 3:**
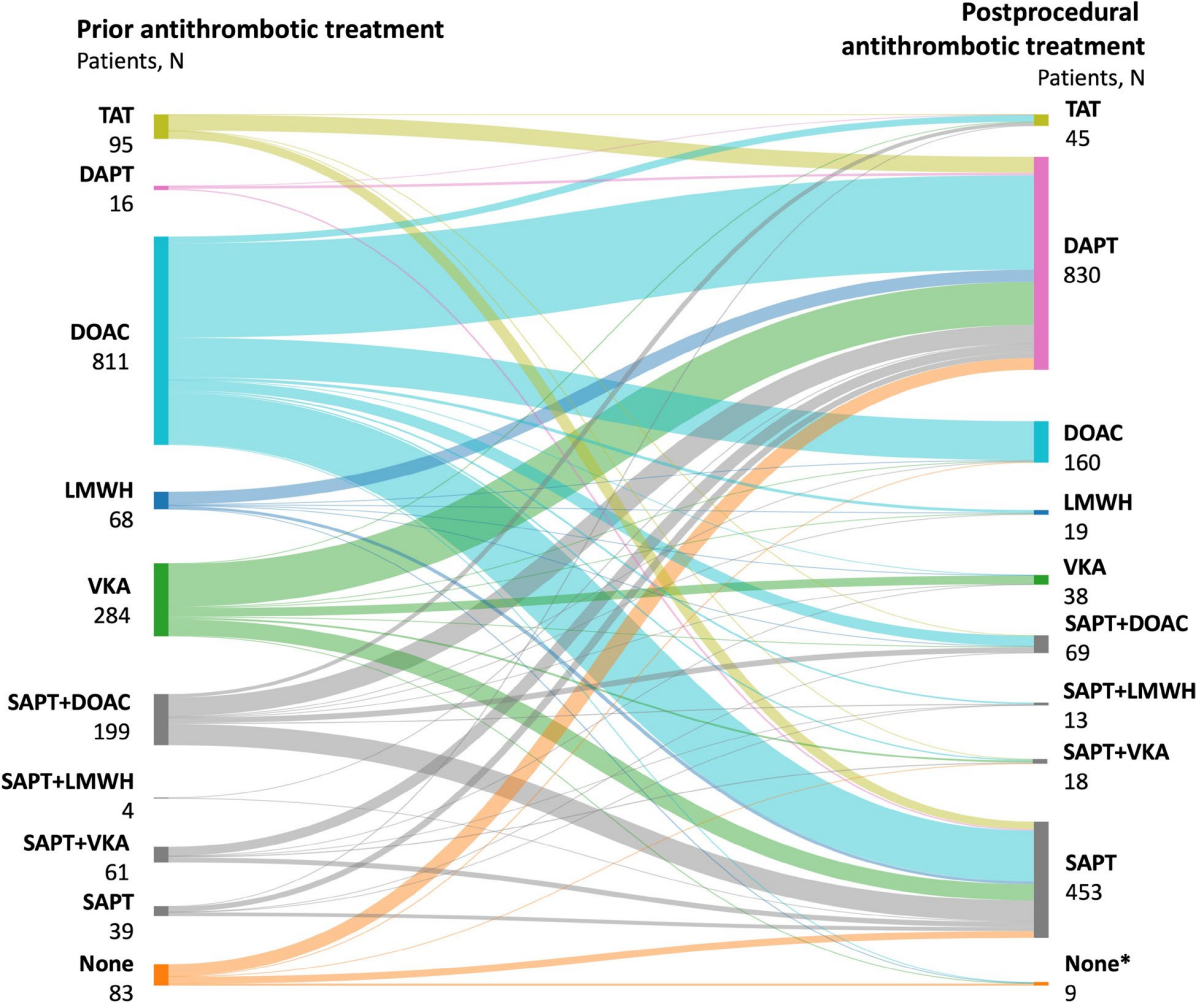
Modification of antithrombotic regimen after the procedure. DAPT indicates dual antiplatelet therapy; DOAC, direct oral anticoagulant; LMWH, low‐molecular‐weight heparin; SAPT, single antiplatelet therapy; TAT, triple antithrombotic therapy; and VKA, vitamin K antagonist. *AtriClip does not require antithrombotic therapy.

An illustrative distribution of changes in antithrombotic therapy is presented in Figure 3.

### Primary Outcomes

The technical success rate was 95.5%, with the most common causes of failure being residual leak (2.2%) and tamponade (1.4%). The procedural success rate was 90.5%. The most frequent factors of procedural failure were vascular complications (3.7%), periprocedural death (1.1%), and major bleeding (0.7%). Among the 19 periprocedural deaths, the leading causes were cardiac tamponade (5 cases [26.3%]) and major bleeding (4 cases [21.1%]). Urgent cardiac surgery was required in 9 patients, including the need for device removal in 5 cases (55.6%). A detailed description of the primary outcomes is presented in Table 2. No statistically significant differences were observed among the occluders in technical and procedural success rates, as shown in Table 2. Detailed results of the univariate analysis are available in Tables S5 and S6.

**Table 2:** Primary Outcomes Overall and on the Basis of the Type of Device Used

	Overall, %	Amplatzer Amulet, %	AtriClip, %	LARIAT, %	Lambre, %	Omega, %	Watchman, %	Watchman FLX, %	*P* value
Technical success	95.5	95.8	94.5	96.6	94.4	91.4	94.5	96.1	0.777
Device embolization	0.2	0.2	0	0	0	0	0.7	0	0.455
Residual leak	2.2	1.2	0	0	2.8	8.6	2.7	2.6	0.048
Surgical device removal	0.3	0.6	0	0	0	0	0.3	0.2	0.853
Percutaneous device removal	0.5	0.8	0	0	0	0	0	0.6	0.706
Urgent cardiosurgery intervention	0.5	0.8	5.5	0	1.4	0	0.3	0	<0.001
Tamponade	1.4	1.6	0	3.4	4.2	0	1.7	0.8	0.159
Procedural success	90.5	92.2	90.9	84.7	93.1	85.7	86.3	91.7	0.05
Vascular complications	3.7	2.8	0	6.8	1.4	2.9	6.2	3.7	0.091
Thromboembolism event	0.5	0.6	1.8	1.7	0	0	0.7	0.2	0.398
Stroke	0.4	0.2	1.8	0	0	2.9	0.3	0.5	0.191
Air embolism	0.1	0	0	0	0	0	0.3	0	0.582
Periprocedural death	1.1	0.8	1.8	3.4	1.4	0	2.1	0.8	0.342
Major bleeding	0.7	0.4	0	0	1.4	2.9	0.7	0.9	0.596

No statistically significant differences were observed in the frequency of primary outcomes after dividing the analyzed period into 2 time intervals. Year‐by‐year analyses demonstrated stable technical and procedural success rates, whereas the incidence of major bleeding and periprocedural death varied significantly over time (*P*<0.001 and *P*=0.003, respectively). Notably, these adverse events showed lower rates in more recent years compared with earlier peaks, albeit with marked interannual variability. Temporal trends in the frequency of primary outcomes and in the use of individual devices are presented in Tables S7 through S9.

## Discussion

This study represents one of the largest European cohorts evaluating the outcomes of stand‐alone LAAO for thromboembolism prevention in patients with nonvalvular AF. Our findings demonstrate that the stand‐alone LAAO procedure is associated with a high technical (95.5%) and procedural (90.5%) success rate, regardless of the device type, reflecting the advances in device technology and operator expertise. Second, we observed a reduction in the use of antiplatelet and anticoagulant therapy in patients undergoing LAAO, highlighting the procedure's potential to minimize long‐term pharmacological burden and associated bleeding risks. These results reinforce the growing evidence supporting LAAO as a viable and effective alternative to long‐term OAC, especially in patients with contraindications or intolerance to anticoagulant therapy. Two general problems are observed in many patients with AF receiving OAC. The first is an inadequate level of anticoagulation due to poor compliance, leading to increased stroke risk, especially for DOAC. The second is an induction of bleeding, which leads to discontinuing the therapy. One real‐world study revealed that ≈30% of patients with nonvalvular AF discontinue OAC in the first 2 years after therapy admission.^16^ The most frequently documented reasons for discontinuing anticoagulation were bleeding (13%) and infrequent AF paroxysms (14%).^17^ In the United States, analysis of MarketScan data (12 129 patients) revealed that up to 47% of patients with nonvalvular AF discontinued anticoagulation therapy in an average of 120 days, most commonly due to bleeding.^16^ Hence, a reliable management option is needed for these patients. Previous real‐world studies, although based on smaller patient cohorts and using a narrower spectrum of devices, demonstrate technical and procedural success rates comparable with the outcomes achieved in our study. One Belgian study involving 457 patients undergoing transcatheter LAAO with the use of either the Watchman or Amplatzer cardiac plug/Amulet device reported a technical success rate of 97.1% and a procedural success rate of 95.9%.^18^ The EWOLUTION (Registry on WATCHMAN Outcomes in Real‐Life Utilization) registry, which included 1025 patients undergoing transcatheter LAAO with the Watchman device, demonstrated technical and procedural success rates of 98.5% and 97.3%, respectively.^19^

In the ITALIAN‐FLX registry, which included 772 patients, there was a technical success rate of 100% and a procedural success rate of 97.3%.^20^ Large randomized clinical trials have shown technical success rates that, while comparable, remain lower: 88% in PROTECT‐AF and 90% in PRAGUE‐17.^12^, ^21^ The high rates of technical and procedural success reported in both real‐world studies, such as ours, and in randomized clinical trials underscore the safety and feasibility of transcatheter LAAO. In addition, a number of studies showed good performance of external epicardial occlusion devices both in animal studies and clinically implanted concomitantly to open heart surgery and thoracoscopic ablation, revealing no safety issues and nearly 100% of LAA closure with no pouch and stable long‐term results.^22^, ^23^, ^24^, ^25^, ^26^, ^27^, ^28^ Different epicardial (AtriClip) and endocardial devices (Watchman, Amulet), are in various stages of development or clinical use with good results in experienced centers. Some doubts about LAAO effectiveness have been formulated in terms of transcatheter techniques.^29^, ^30^, ^31^ There is a debate whether it is related to acute complications, need for bridging anticoagulation, mid‐ and long‐term device leakage, or thromboembolism originating from other parts of the left atrium.^32^, ^33^ Epicardial clips are able to avoid those weaknesses, while limitations associated with the thoracoscopic intervention, such as the need for short induction and intubation, severely depressed lung function, and previous surgery with left pleural cavity opening, are self‐evident. The optimal antithrombotic regimen following LAAO remains debatable. Despite growing experience with the procedure and, therefore, increasing implantation success rates, there is still no universally accepted postimplantation protocol. Various strategies have been proposed, ranging from DAPT to short‐term OAC, depending on patient bleeding risk profiles. The current evidence on the optimal antithrombotic strategy following LAAO remains conflicting, especially since studies include patients with widely varying bleeding risks and use inconsistent regimens. There is no general consensus on the most effective/safe approach, and clinical practice can vary significantly between centers.^34^ While some studies support the use of low‐dose DOACs for reducing thromboembolic and bleeding events, others report no significant advantage over simplified regimens such as SAPT or even no therapy.^35^ A network meta‐analysis of 41 studies^36^ found that DOAC monotherapy was associated with the lowest rates of thromboembolic events and the highest risk of major bleeding, while DOAC treatment was superior to VKA in reducing all‐cause death by 61%, and DAPT was associated with 50% lower risk of thromboembolic events compared with SAPT. Clinical trial evidence comparing antithrombotic regimes is limited. The ADALA (Low‐Dose Direct Oral Anticoagulation Versus Dual Antiplatelet Therapy After Left Atrial Appendage Occlusion) trial identified that low‐dose DOAC, in the form of apixaban 2.5 mg twice daily, was a superior therapeutic option, reducing the risk of the primary end point (major bleeding, thromboembolic events, and device‐related thrombosis) by 81% at 3‐month follow‐up.^37^ Conversely, there are some observational data. For example, findings from the LOGIC (Left Atrial Appendage Occlusion in Patients With Gastrointestinal or Intracranial Bleeding) registry demonstrated no significant differences in overall death, cardiovascular death, thromboembolic events, or bleeding risk between patients receiving SAPT or no antithrombotic therapy and those treated with standard antithrombotic regimens.^38^ In the EWOLUTION study, the use of DAPT was comparable (60.8% versus 50% in our cohort), while SAPT was less common (6.9% versus 27.3% in our study).^19^ In contrast, John et al reported DAPT use in 72% of their patients, with limited use of DOACs (3.7% versus 9.8% in our cohort).^18^ In the SALAMANDER registry, discharge antithrombotic therapy varied not only between centers but also at the operator level (as the choice of postprocedural regimen was left to the operator's discretion). This likely reflects real‐world decision making that is strongly shaped by the individual patient's clinical profile, procedure‐related anatomic considerations, and implant quality (optimal anatomic sealing with stable device positioning). In many cases, the predominant driver is a bleeding‐prone phenotype (frequent prior major bleeding, particularly gastrointestinal or intracranial), in whom even short‐term OAC may be considered unacceptable, favoring DAPT or SAPT. Conversely, a distinct subgroup undergoes LAAO due to perceived OAC failure, that is, thromboembolic events (including stroke) occurring despite anticoagulation, which may prompt some operators to prefer a period of OAC or DOAC‐based therapy after the procedure, even for long‐term use. These patient‐level contrasts, combined with center/operator‐specific decisions, plausibly explain the observed heterogeneity in post‐LAAO regimens. This heterogeneity also mirrors differences between landmark trials. In PROTECT AF and PREVAIL, the postimplant protocol was anchored in short‐term warfarin plus aspirin followed by a transition to antiplatelet therapy, reflecting a strategy that assumes temporary anticoagulation is feasible in many patients. In contrast, PRAGUE‐17 enrolled a high‐risk population and used a more antiplatelet‐forward post‐LAAO approach, which is closer to the DAPT‐dominant practice seen in contemporary bleeding‐driven cohorts such as SALAMANDER.^10^, ^11^, ^12^

Device selection (endocardial versus epicardial) for LAAO is usually determined by LAA anatomy, bleeding risk profile, feasibility of antithrombotic therapy, and institutional expertise.^8^ Endocardial systems require transseptal access and implantation within the LAA lumen, necessitating at least a temporary course of anticoagulant or antiplatelet therapy. In contrast, epicardial techniques achieve LAA exclusion without leaving permanent material within the LAA. This fundamental distinction often allows simplification or avoidance of long‐term anticoagulation.^39^ Moreover, epicardial systems require pericardial access or surgical exposure, which introduces procedure‐specific risks such as pericardial bleeding, effusion, tamponade, and injury to adjacent structures.^8^ Endocardial devices, by contrast, are implanted via a fully percutaneous transseptal approach and are therefore associated with a lower immediate procedural risk profile. Their periprocedural complications are more commonly related to transseptal puncture, device embolization, or access‐site bleeding.^40^ In clinical practice, these differences directly influence patient selection and the individualization of periprocedural and postprocedural management strategies.

## Limitations

The SALAMANDER registry is an observational, real‐world multicenter cohort study. The AF population has been recruited across 2 countries (Poland and Italy), but as with any observational cohort study, there are limitations, including common limitations to observational cohort studies, inadequacy of data recording/capture, and selection bias. Despite selecting high‐volume reference centers to ensure uniform procedural quality, certain data elements were not collected systematically across sites. In this context, a notable example is the lack of detailed information on imaging modalities used during percutaneous LAAO procedures, which reflects the inherent challenges of comprehensive data acquisition in large observational registries. The large overall study cohort is a strength of this study; however, the marked imbalance in sample sizes between device groups limits the ability to reliably compare outcomes, particularly rare events such as tamponade, and therefore warrants cautious interpretation. Finally, as the present cohort is derived exclusively from European populations, the generalizability of these findings to other regions may be limited, given the known ethnic differences in AF‐related complication (ie, stroke and bleeding),^41^, ^42^ LAA morphology,^43^ and the so‐called “East Asian” paradox in relation to antithrombotic therapy effectiveness and safety^44^; therefore, similarly large prospective cohorts from different populations globally are warranted.

## Conclusions

In one of the largest European cohorts, we show that the stand‐alone LAAO procedure is associated with a high technical and procedural success rate, with potentially comparable outcomes across device types. The reduction in the use of antiplatelet and anticoagulant therapy in patients undergoing LAAO reduces long‐term OAC use and associated bleeding risks.

## Sources of Funding

The SALAMANDER study was initiated and funded by the Scientific Platform of the Polish Cardiac Society. The funding body had no role in study design, data collection, data analysis, data interpretation, or article writing.

## Disclosures

Dr Lewicki has consulted and lectured for Abbott and Boston Scientific outside the present work. Dr Grasso has consulted, lectured, and served as an advisory board member for Abbott, Boston Scientific, and Edwards outside the present work. Dr Grygier has consulted, lectured, and served as an advisory board member for Boston Scientific outside the present work. Dr Smolka has consulted and lectured for Abbott and Boston Scientific outside the present work. Dr Kapłon‐Cieślicka has consulted, served as an advisory board member, received honoraria for lectures, and received travel grants from Adamed, AstraZeneca, Bayer, Bausch Health, Boehringer Ingelheim, KRKA, Pfizer, Polpharma, and Servier outside the present work. Dr Kralisz consulted and lectured for Boston Scientific outside the present work. Dr Waciński has consulted, lectured, and served as an advisory board member for Philips outside the present work. The remaining authors have no disclosures to report.

## Supporting information

Data S1
Tables S1–S9
