# Supplementary material for: Primary Results of the SALAMANDER Registry: A Multicenter Observational Cohort Study
Source: J Am Heart Assoc. 2026 May 14;15(10):e046625. doi: 10.1161/JAHA.125.046625 (PMC13279340; doi:10.1161/JAHA.125.046625)
Supplement: Supplementary file 1 — Data S1 Tables S1–S9 [file JAH3-15-e046625-s001.pdf]

Supplemental Material

Data S1. Supplemental Methods

Baseline characteristics encompassed patient age, sex, body mass index, and arrhythmia subtype (AF or AFl). Comorbid conditions were carefully documented, including congestive heart failure, hypertension, diabetes mellitus, chronic kidney disease, coronary artery disease, peripheral artery disease, sleep apnea, and functional impairment as assessed by EHRA and NYHA classification systems. The risk of thromboembolic events and bleeding was assessed using the CHA_2DS_2-VASc and HAS-BLED scoring systems, respectively, supplemented with laboratory parameters such as estimated glomerular filtration rate (GFR), haemoglobin concentration, and platelet count. A detailed medical history was obtained to capture previous major bleeding events - including gastrointestinal bleeding, haemorrhagic stroke, and epistaxis - as well as known contraindications to oral anticoagulation, such as congenital coagulopathies, thrombocytopenia, dialysis dependency, or drug hypersensitivity. Imaging modalities, including transoesophageal echocardiography and computed tomography, were employed to characterize left atrial appendage morphology, size, and peak flow velocity. These measurements supported the individualized selection of occlusion devices. Procedural data included the type of LAAO device used, anaesthetic strategy, total procedural duration, and device-specific technical metrics such as implantation depth, degree of compression, presence of residual leaks, and need for device recapture. Procedural complications, particularly device embolization or surgical device retrieval, were also recorded. Postprocedural pharmacotherapy included antiplatelet and anticoagulant agents such as aspirin, clopidogrel, ticagrelor, VKA, DOAC, and low-molecular-weight heparins. The type and duration of postprocedural therapy were left to the discretion of the treating physicians. This structured and detailed dataset provides a robust foundation for subsequent comparative analyses, contributing meaningful insights into the safety, efficacy, and optimization of LAAO in contemporary clinical practice.

Table S1. STROBE checklist.

	Item No	Recommendation	Page number
**Title and abstract**	1	(*a*) Indicate the study's design with a commonly used term in the title or the abstract	1
		(*b*) Provide in the abstract an informative and balanced summary of what was done and what was found	2
**Introduction**			
Background/rationale	2	Explain the scientific background and rationale for the investigation being reported	5
Objectives	3	State specific objectives, including any prespecified hypotheses	6
**Methods**			
Study design	4	Present key elements of study design early in the paper	7, Figure 1
Setting	5	Describe the setting, locations, and relevant dates, including periods of recruitment, exposure, follow-up, and data collection	7-9, Table 1
Participants	6	(*a*) *Cohort study*—Give the eligibility criteria, and the sources and methods of selection of participants. Describe methods of follow-up<br>*Case-control study*—Give the eligibility criteria, and the sources and methods of case ascertainment and control selection. Give the rationale for the choice of cases and controls<br>*Cross-sectional study*—Give the eligibility criteria, and the sources and methods of selection of participants	6-9
		(*b*) *Cohort study*—For matched studies, give matching criteria and number of exposed and unexposed<br>*Case-control study*—For matched studies, give matching criteria and the number of controls per case	N/A
Variables	7	Clearly define all outcomes, exposures, predictors, potential confounders, and effect modifiers. Give diagnostic criteria, if applicable	6-8
Data sources/ measurement	8*	For each variable of interest, give sources of data and details of methods of assessment (measurement). Describe comparability of assessment methods if there is more than one group	6-8
Bias	9	Describe any efforts to address potential sources of bias	7-8

Study size	10	Explain how the study size was arrived at	7, 15
Quantitative variables	11	Explain how quantitative variables were handled in the analyses. If applicable, describe which groupings were chosen and why	8
Statistical methods	12	(*a*) Describe all statistical methods, including those used to control for confounding	8
		(*b*) Describe any methods used to examine subgroups and interactions	8
		(*c*) Explain how missing data were addressed	10
		(*d*) *Cohort study*—If applicable, explain how loss to follow-up was addressed<br>*Case-control study*—If applicable, explain how matching of cases and controls was addressed<br>*Cross-sectional study*—If applicable, describe analytical methods taking account of sampling strategy	7-8
		(*e*) Describe any sensitivity analyses	8
**Results**			
Participants	13*	(a) Report numbers of individuals at each stage of study—eg numbers potentially eligible, examined for eligibility, confirmed eligible, included in the study, completing follow-up, and analysed	7-8, Figure 1
		(b) Give reasons for non-participation at each stage	7, Figure 1
		(c) Consider use of a flow diagram	Figure 1
Descriptive data	14*	(a) Give characteristics of study participants (eg demographic, clinical, social) and information on exposures and potential confounders	8-9, Table 1
		(b) Indicate number of participants with missing data for each variable of interest	Table 1
		(c) *Cohort study*—Summarise follow-up time (eg, average and total amount)	Table 1
Outcome data	15*	*Cohort study*—Report numbers of outcome events or summary measures over time	10, Table 2
		Case-control study—Report numbers in each exposure category, or summary measures of exposure	N/A
		Cross-sectional study—Report numbers of outcome events or summary measures	N/A
Main results	16	(*a*) Give unadjusted estimates and, if applicable, confounder-adjusted estimates and their precision (eg, 95% confidence interval). Make clear which confounders were adjusted for and why they were included	10, Supplementary Table 5-6
		(*b*) Report category boundaries when continuous variables were categorized	N/A

		(*c*) If relevant, consider translating estimates of relative risk into absolute risk for a meaningful time period	N/A
Other analyses	17	Report other analyses done—eg analyses of subgroups and interactions, and sensitivity analyses	9-10, Supplementary Table 7-9
**Discussion**			
Key results	18	Summarise key results with reference to study objectives	10
Limitations	19	Discuss limitations of the study, taking into account sources of potential bias or imprecision. Discuss both direction and magnitude of any potential bias	13-14
Interpretation	20	Give a cautious overall interpretation of results considering objectives, limitations, multiplicity of analyses, results from similar studies, and other relevant evidence	11-13
Generalisability	21	Discuss the generalisability (external validity) of the study results	11-13
**Other information**			
Funding	22	Give the source of funding and the role of the funders for the present study and, if applicable, for the original study on which the present article is based	14

Table S2. Study sites.

Site	Amplatzer	AtriClip	Lambre	LARIAT	OMEGA	Watchman	Watchman FLX	Total
1st Department of Cardiology, Medical University of Silesia, Katowice, Poland	53					1		54
3rd Department of Cardiology, Medical University of Silesia, Katowice, Poland	0					176	106	282
Clinical Department of Cardiac Surgery, Central Clinical Hospital of the Ministry of the Interior and Administration in Warsaw, Warsaw, Poland	0	55						55
Department of Cardiology and Interventional Angiology, Specialist Hospital in Wejherowo, Wejherowo, Poland	22						12	34
Department of Cardiology, Catania University Hospital, Catania, Italy	11				32	19	203	265
Department of Cardiology, Medical University of Gdańsk, Gdańsk, Poland	119						6	125
Department of Cardiology, Medical University of Lodz, Lodz, Poland	9		37			1		47
Department of Cardiology, Nicolaus Copernicus University, Bydgoszcz, Poland	6						107	113
Department of Cardiology, Ospedale Sant'Andrea, Vercelli,, Italy	8		12		3		13	36
Department of Cardiology, Poznan University of Medical Sciences, Poznań, Poland	5						53	58
Department of Cardiology, Specialist Hospital in Grudziadz, Grudziadz, Poland.	0		22	59				81

Department of Cardiology, University of Opole, Opole, Poland	4						10	14
Department of Cardiology, University of Turin, Turin, Italy	70					9	32	111
Department of Invasive Cardiology, Medical University of Białystok, Bialystok, Poland	18					61	48	127
Division of Cardiology, Parma University Hospital, University of Parma, Parma, Italy	140		1				13	154
Institute of Heart Diseases, Wroclaw Medical University, Wroclaw, Poland	35					24	45	104
Total	500	55	72	59	35	291	649	1660

Table S3. Comparison of patient characteristics according to the implanted device.

Variable	Amplatzer Amulet, %	AtriClip, %	LARIAT, %	Lambre, %	OMEGA, %	Watchman, %	Watchman FLX, %
Age, years Me (1Q-3Q)	76 (71-81)	73 (67-78)	72 (67-77.5)	76.5 (71.8-81)	80 (74.5-83)	75 (68-79.5)	76 (70-81)
Female, %	38.4	41.8	35.6	40.3	34.3	37.8	37.5
BMI Me (1Q-3Q)	26.5 (24.2-29.6)	27.1 (24.8-28.8)	N/D	27.6 (24.5-30.6)	25.6 (23.1-28.4)	27.8 (24.9-31.2)	27.3 (24.4-30.3)
LVEF, % Me (1Q-3Q)	55 (50-60)	47 (40-55)	50 (40-59)	55 (47-58)	55 (50-55)	50 (46.5-60)	55 (45-58)
HF, %	43.6	90.9	39	72.2	20	42.6	45.8
Hypertension, %	87.8	90.9	100	94.4	88.6	93.8	88.6
Diabetes, %	32.6	20	44.1	37.5	37.1	26.8	37.7
CKD, %	47	40	0	41.7	60	44	50.6
Coronary artery disease, %	40.8	34.5	0	20.8	22.9	44	38.9
Prior MI, % (N)	24.2	7.3	0	11.1	11.4	24.4	20.2
Peripheral artery disease, %	13.8	10.9	69.5	23.6	17.1	18.2	18.4
Carotid artery disease, %	15.6	3.6	0	2.8	8.6	5.8	12
Lung diseases, %	6.4	3.6	0	4.2	5.7	7.9	6.5
Baseline GFR ml/min/1.73m2, Me (1Q-3Q)	61 (42.1-76)	65.5 (52-75)	N/D	56 (42-71.8)	44 (27-67)	62 (47-79)	60 (40-78)
Baseline haemoglobin g/dl, Me (1Q-3Q)	12 (11-13.8)	13.1 (11.7-14.4)	N/D	12.4 (11-14)	10.1 (10.1-10.1)	13 (11.9-14)	12 (11-13.8)
Baseline PLT, Me (1Q-3Q)	208 (166.5-254)	201 (161.5-238.5)	N/D	200 (162-254)	156 (156-178.5)	192 (154-237.8)	205 (164-256.8)

Mitral stenosis (≥ moderate), %	0.6	0	N/D	2.8	0	0.3	0.5
Mitral regurgitation (≥ moderate), %	16.6	43.6	N/D	11.1	0	22.3	25.5
Aortic stenosis (≥ moderate), %	3.4	0	N/D	5.6	20	3.4	9.6
Aortic regurgitation (≥ moderate), %	2.8	1.8	N/D	1.4	0	3.8	5.1

Abbreviations: BMI, body mass index; CKD, chronic kidney disease; GFR, glomerular filtration rate; LVEF, left ventricular ejection fraction; Me, median; MI, myocardial infarction; PLT, platelet count; Q, quartile.

Table S4. Left atrial appendage characteristics.

	**Observations**	**Values**
**LAA type**		
Chicken wing, N (%)	1020	648 (63.5%)
Cactus, N (%)	1020	89 (8.7%)
Windsock, N (%)	1020	138 (13.5%)
Cauliflower, N (%)	1020	128 (12.5%)
Other, N (%)	1020	19 (1.9%)
**LAA parameters**		
LAA diameter (mm), Me (1Q-3Q)	691	21 (18-23)
LAA length (mm), Me (1Q-3Q)	344	27 (21-32)
LAA peak velocity (cm/s), Me (1Q-3Q)	76	35 (25.5-44)
CT LAA diameter (mm), Me (1Q-3Q)	95	22 (19-24)
CT LAA length (mm), Me (1Q-3Q)	25	42 (40-50)

Abbreviations: CT, Computed Tomography; LAA, Left Atrial Appendage; Me, Median; N, Number; Q, Quartile.

Table S5. Univariate analysis of predictors of technical success.

Variable	OR	95% CI	p-value
Age	0.996	0.968-1.024	0.764
Sex	1.288	0.784-2.118	0.318
BMI	1.018	0.966-1.073	0.512
CHF	0.686	0.429-1.097	0.116
Hypertension	1.432	0.721-2.846	0.305
Diabetes	0.74	0.46-1.191	0.215
CKD	0.846	0.531-1.349	0.483
History of thromboembolism	1.095	0.653-1.836	0.73
Prior ischemic stroke	0.897	0.535-1.506	0.682
Prior hemorrhagic stroke	0.902	0.456-1.787	0.768
Prior systemic embolism	5.165	1.257-21.217	0.023
Vascular disease	1.31	0.782-2.195	0.305
CAD	1.196	0.731-1.957	0.476
Prior MI	1.105	0.61-2.001	0.743
PAD	1.699	0.837-3.449	0.143
Carotid artery disease	4.551	1.107-18.709	0.036
Sleep apnoea	0.321	0.072-1.437	0.137
Lung disease	0.624	0.279-1.395	0.25
CHA2DS2VA score	0.965	0.829-1.123	0.647
HAS-BLED score	0.902	0.734-1.11	0.33
Baseline GFR	1	0.99-1.01	0.972
Baseline haemoglobin	1.006	0.884-1.146	0.924
Baseline PLT	1	0.997-1.003	0.999
Mitral stenosis (≥ moderate)	0.16	0.033-0.782	0.024

Mitral regurgitation (≥ moderate)	1.13	0.624-2.048	0.686
Aortic stenosis (≥ moderate)	0.44	0.212-0.911	0.027
Aortic regurgitation (≥ moderate)	0.496	0.192-1.278	0.146
CHA2DS2VA score >=5	1.239	0.644-2.38	0.521
HAS-BLED score >=3	0.56	0.24-1.305	0.179
Coagulopathies	1.599	0.216-11.845	0.646
Failure of oral anticoagulation	1.424	0.44-4.609	0.556
Dialysis	0.736	0.261-2.078	0.563
prior significant bleeding	0.523	0.238-1.152	0.108
LAA diameter	0.936	0.842-1.04	0.219
LAA lenght	0.982	0.923-1.044	0.559
Anaesthesia with intubation	1.531	0.891-2.63	0.123
General anaesthesia	0.964	0.602-1.544	0.88
Time of procedure	0.992	0.984-1	0.046

Abbreviations: BMI, body mass index; CAD, coronary artery disease; CHA2DS2VA, congestive heart failure, hypertension, age ≥75 years, diabetes mellitus, prior stroke or TIA or thromboembolism, vascular disease, age 65–74 years, and sex category (female); CHF, congestive heart failure; CKD, chronic kidney disease; GFR, glomerular filtration rate; HAS-BLED, hypertension, abnormal liver/renal function, stroke, bleeding history or predisposition, labile INR, elderly (age >65), drugs/alcohol concomitantly; INR, international normalized ratio; LVEF, left ventricular ejection fraction; MI, myocardial infarction; PAD, peripheral artery disease; PLT, platelet count; TIA, transient ischemic attack

Table S6. Univariate analysis of predictors of procedural success.

Variable	OR	95% CI	p-value
Age	0.992	0.972-1.012	0.426
Sex	1.018	0.725-1.429	0.92
BMI	1.037	0.997-1.079	0.07
CHF	0.732	0.526-1.017	0.063
Hypertension	1.357	0.824-2.235	0.231
Diabetes	0.888	0.63-1.25	0.496
CKD	1.007	0.724-1.401	0.965
History of thromboembolism	1.013	0.707-1.45	0.944
Prior ischemic stroke	0.942	0.651-1.362	0.751
Prior hemorrhagic stroke	0.909	0.56-1.476	0.7
Prior systemic embolism	1.921	1.021-3.611	0.043
Vascular disease	1.109	0.78-1.577	0.565
CAD	1.136	0.805-1.603	0.467
Prior MI	1.325	0.855-2.055	0.209
PAD	0.931	0.615-1.408	0.733
Carotid artery disease	2.111	1.057-4.215	0.034
Sleep apnoea	0.448	0.126-1.589	0.214
Lung disease	0.651	0.361-1.172	0.152
CHA2DS2VA score	0.95	0.854-1.058	0.35
HAS-BLED score	0.89	0.769-1.031	0.12
Baseline GFR	1	0.993-1.007	0.956
Baseline haemoglobin	0.947	0.863-1.038	0.245
Baseline PLT	1.002	1-1.005	0.086
Mitral stenosis (≥ moderate)	0.363	0.075-1.761	0.208
Mitral regurgitation (≥ moderate)	1.027	0.683-1.545	0.896

Aortic stenosis (≥ moderate)	0.567	0.319-1.009	0.054
Aortic regurgitation (≥ moderate)	0.783	0.35-1.754	0.552
CHA2DS2VA score >=5	1.088	0.7-1.692	0.707
HAS-BLED score >=3	0.61	0.345-1.076	0.088
Coagulopathies	1.74	0.414-7.32	0.45
Failure of oral anticoagulation	2.408	0.872-6.647	0.09
Dialysis	1.083	0.461-2.545	0.855
prior significant bleeding	0.475	0.27-0.835	0.01
LAA diameter	0.929	0.864-0.999	0.047
LAA lenght	0.986	0.943-1.032	0.549
Anaesthesia with intubation	1.269	0.881-1.827	0.201
General anaesthesia	0.721	0.513-1.012	0.059
Time of procedure	0.997	0.991-1.003	0.35

Abbreviations: BMI, body mass index; CAD, coronary artery disease; CHA2DS2VA, congestive heart failure, hypertension, age ≥75 years, diabetes mellitus, prior stroke or TIA or thromboembolism, vascular disease, age 65–74 years, and sex category (female); CHF, congestive heart failure; CKD, chronic kidney disease; GFR, glomerular filtration rate; HAS-BLED, hypertension, abnormal liver/renal function, stroke, bleeding history or predisposition, labile INR, elderly (age >65), drugs/alcohol concomitantly; INR, international normalized ratio; LVEF, left ventricular ejection fraction; MI, myocardial infarction; PAD, peripheral artery disease; PLT, platelet count; TIA, transient ischemic attack

Table S7. Rates of primary outcomes across two time periods (2010-2020 and 2021-2024).

	**2010-2020**	**2021-2024**	**p-value**
**Observations**	788	872	N/A
**Technical success**	753 (95.6%)	833 (95.5%)	0.976
Device embolization	3 (0.4%)	0 (0%)	0.068
Residual leak	14 (1.8%)	22 (2.5%)	0.297
Surgical device removal	3 (0.4%)	2 (0.2%)	0.574
Percutaneous device removal	2 (0.3%)	6 (0.7%)	0.202
Urgent cardiosurgery intervention	7 (0.9%)	2 (0.2%)	0.068
Tamponade	13 (1.6%)	10 (1.1%)	0.381
**Procedural success**	705 (89.5%)	798 (91.5%)	0.155
Vascular complications	33 (4.2%)	29 (3.3%)	0.355
Thromboembolism event	5 (0.6%)	3 (0.3%)	0.393
Stroke	3 (0.4%)	4 (0.5%)	0.807
Air embolism	1 (0.1%)	0 (0%)	0.293
Periprocedural death	13 (1.6%)	6 (0.7%)	0.066
Major bleeding	8 (1%)	4 (0.5%)	0.181

Table S8. Annual rates of primary outcomes.

	2010	2011	2012	2013	2014	2015	2016	2017	2018	2019	2020	2021	2022	2023	2024	p-value
**Observations**	3	5	6	9	38	104	98	105	115	143	162	240	304	260	68	N/A
**Technical success**	3 (100%)	4 (80%)	6 (100%)	9 (100%)	37 (97.4%)	100 (96.2%)	96 (98%)	100 (95.2%)	108 (93.9%)	136 (95.1%)	154 (95.1%)	231 (96.3%)	289 (95.1%)	251 (96.5%)	62 (91.2%)	0.733
Device embolization	0 (0%)	0 (0%)	0 (0%)	0 (0%)	0 (0%)	1 (1%)	1 (1%)	1 (1%)	0 (0%)	0 (0%)	0 (0%)	0 (0%)	0 (0%)	0 (0%)	0 (0%)	0.506
Residual leak	0 (0%)	0 (0%)	0 (0%)	0 (0%)	1 (2.6%)	1 (1%)	0 (0%)	1 (1%)	5 (4.3%)	2 (1.4%)	4 (2.5%)	5 (2.1%)	8 (2.6%)	3 (1.2%)	6 (8.8%)	0.061
Surgical device removal	0 (0%)	0 (0%)	0 (0%)	0 (0%)	0 (0%)	0 (0%)	1 (1%)	0 (0%)	0 (0%)	1 (0.7%)	1 (0.6%)	0 (0%)	1 (0.3%)	1 (0.4%)	0 (0%)	0.984
Percutaneous device removal	0 (0%)	0 (0%)	0 (0%)	0 (0%)	0 (0%)	0 (0%)	1 (1%)	0 (0%)	0 (0%)	0 (0%)	1 (0.6%)	2 (0.8%)	4 (1.3%)	0 (0%)	0 (0%)	0.775
Urgent cardiosurgery intervention	0 (0%)	0 (0%)	0 (0%)	0 (0%)	0 (0%)	0 (0%)	1 (1%)	0 (0%)	1 (0.9%)	4 (2.8%)	1 (0.6%)	0 (0%)	0 (0%)	2 (0.8%)	0 (0%)	0.157
Tamponade	0 (0%)	1 (20%)	0 (0%)	0 (0%)	0 (0%)	2 (1.9%)	0 (0%)	3 (2.9%)	2 (1.7%)	4 (2.8%)	1 (0.6%)	3 (1.3%)	3 (1%)	4 (1.5%)	0 (0%)	0.102
**Procedural success**	3 (100%)	4 (80%)	6 (100%)	9 (100%)	33 (86.8%)	90 (86.5%)	91 (92.9%)	86 (81.9%)	104 (90.4%)	131 (91.6%)	148 (91.4%)	223 (92.9%)	277 (91.1%)	239 (91.9%)	59 (86.8%)	0.182
Vascular complications	0 (0%)	0 (0%)	0 (0%)	0 (0%)	4 (10.5%)	6 (5.8%)	5 (5.1%)	9 (8.6%)	3 (2.6%)	4 (2.8%)	2 (1.2%)	8 (3.3%)	13 (4.3%)	6 (2.3%)	2 (2.9%)	0.136
Thromboembolism event	0 (0%)	0 (0%)	0 (0%)	0 (0%)	0 (0%)	2 (1.9%)	0 (0%)	1 (1%)	1 (0.9%)	0 (0%)	1 (0.6%)	1 (0.4%)	0 (0%)	2 (0.8%)	0 (0%)	0.822
Stroke	0 (0%)	0 (0%)	0 (0%)	0 (0%)	0 (0%)	0 (0%)	0 (0%)	2 (1.9%)	0 (0%)	0 (0%)	1 (0.6%)	0 (0%)	1 (0.3%)	2 (0.8%)	1 (1.5%)	0.649
Air embolism	0 (0%)	0 (0%)	0 (0%)	0 (0%)	0 (0%)	1 (1%)	0 (0%)	0 (0%)	0 (0%)	0 (0%)	0 (0%)	0 (0%)	0 (0%)	0 (0%)	0 (0%)	0.380

Periprocedural death	0 (0%)	1 (20%)	0 (0%)	0 (0%)	0 (0%)	2 (1.9%)	0 (0%)	4 (3.8%)	0 (0%)	3 (2.1%)	3 (1.9%)	0 (0%)	1 (0.3%)	4 (1.5%)	1 (1.5%)	0.003
Major bleeding	0 (0%)	1 (20%)	0 (0%)	0 (0%)	0 (0%)	0 (0%)	0 (0%)	0 (0%)	0 (0%)	6 (4.2%)	1 (0.6%)	0 (0%)	1 (0.3%)	2 (0.8%)	1 (1.5%)	<0.001

Table S9. Annual trends in implanted device types.

Year	Amplatzer	AtriClip	LARIAT	Lambre	OMEGA	Watchman	Watchman FLX
**2010, N (%)**	3 (0.2%)	0 (0%)	0 (0%)	0 (0%)	0 (0%)	0 (0%)	0 (0%)
**2011, N (%)**	5 (0.3%)	0 (0%)	0 (0%)	0 (0%)	0 (0%)	0 (0%)	0 (0%)
**2012, N (%)**	6 (0.4%)	0 (0%)	0 (0%)	0 (0%)	0 (0%)	0 (0%)	0 (0%)
**2013, N (%)**	9 (0.5%)	0 (0%)	0 (0%)	0 (0%)	0 (0%)	0 (0%)	0 (0%)
**2014, N (%)**	13 (0.8%)	0 (0%)	0 (0%)	0 (0%)	0 (0%)	25 (1.5%)	0 (0%)
**2015, N (%)**	26 (1.6%)	7 (0.4%)	24 (1.4%)	9 (0.5%)	0 (0%)	38 (2.3%)	0 (0%)
**2016, N (%)**	26 (1.6%)	8 (0.5%)	11 (0.7%)	12 (0.7%)	0 (0%)	41 (2.5%)	0 (0%)
**2017, N (%)**	26 (1.6%)	8 (0.5%)	20 (1.2%)	1 (0.1%)	0 (0%)	46 (2.8%)	4 (0.2%)
**2018, N (%)**	31 (1.9%)	8 (0.5%)	4 (0.2%)	6 (0.4%)	0 (0%)	60 (3.6%)	6 (0.4%)

**2019, N (%)**	50 (3%)	3 (0.2%)	0 (0%)	11 (0.7%)	0 (0%)	64 (3.9%)	15 (0.9%)
**2020, N (%)**	60 (3.6%)	4 (0.2%)	0 (0%)	7 (0.4%)	0 (0%)	15 (0.9%)	76 (4.6%)
**2021, N (%)**	82 (4.9%)	1 (0.1%)	0 (0%)	9 (0.5%)	0 (0%)	2 (0.1%)	146 (8.8%)
**2022, N (%)**	91 (5.5%)	6 (0.4%)	0 (0%)	3 (0.2%)	2 (0.1%)	0 (0%)	202 (12.2%)
**2023, N (%)**	63 (3.8%)	10 (0.6%)	0 (0%)	10 (0.6%)	15 (0.9%)	0 (0%)	162 (9.8%)
**2024, N (%)**	9 (0.5%)	0 (0%)	0 (0%)	4 (0.2%)	18 (1.1%)	0 (0%)	37 (2.2%)
**All, N (%)**	500 (30.1%)	55 (3.3%)	59 (3.6%)	72 (4.3%)	35 (2.1%)	291 (17.5%)	648 (39%)
